# 8-Hy­droxy­quinolin-1-ium nitrate

**DOI:** 10.1107/S1600536810039395

**Published:** 2010-10-23

**Authors:** Wan-Sin Loh, Madhukar Hemamalini, Hoong-Kun Fun

**Affiliations:** a X-ray Crystallography Unit, School of Physics, Universiti Sains Malaysia, 11800 USM, Penang, Malaysia

## Abstract

In the title salt, C_9_H_8_NO^+^·NO_3_^−^, the quinoline ring system is essentially planar with a maximum deviation of 0.043 (1) Å. In the crystal, an *R*_2_^2^(7) ring motif is formed by inter­molecular N—H⋯O and C—H⋯O hydrogen bonds between the cation and the anion. In addition, inter­molecular O—H⋯O and C—H⋯O hydrogen bonds link the two ions, generating an *R*_2_^2^(8) ring motif. These sets of ring motifs are further linked into a ribbon along the *a* axis *via* inter­molecular C—H⋯O hydrogen bonds.

## Related literature

For background to and the biological activity of quinoline derivatives, see: Campbell *et al.* (1988 ▶); Markees *et al.* (1970 ▶); Michael (1997 ▶); Morimoto *et al.* (1991 ▶); Reux *et al.* (2009 ▶); Sasaki *et al.* (1998 ▶). For related structures, see: Loh *et al.* (2010*a* ▶,*b* ▶,*c* ▶,*d* ▶). For the stability of the temperature controller used in the data collection, see: Cosier & Glazer (1986 ▶). For bond-length data, see: Allen *et al.* (1987 ▶).
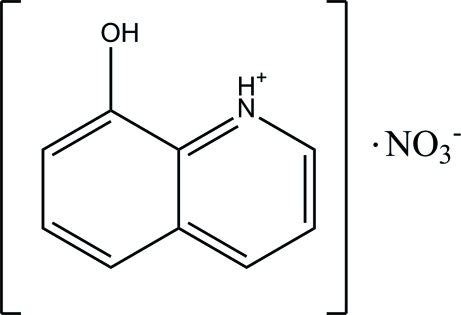


## Experimental

### 

#### Crystal data

C_9_H_8_NO^+^·NO_3_^−^
*M* *_r_* = 208.17
Monoclinic, 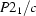

*a* = 11.3186 (2) Å
*b* = 6.7568 (1) Å
*c* = 14.5006 (2) Å
β = 128.882 (1)°
*V* = 863.27 (2) Å^3^
*Z* = 4
Mo *K*α radiation
μ = 0.13 mm^−1^
*T* = 100 K
0.33 × 0.21 × 0.15 mm

#### Data collection

Bruker SMART APEXII CCD area-detector diffractometer
Absorption correction: multi-scan (*SADABS*; Bruker, 2009 ▶) *T*_min_ = 0.959, *T*_max_ = 0.981
9590 measured reflections
1978 independent reflections
1754 reflections with *I* > 2σ(*I*)
*R*_int_ = 0.023

#### Refinement

*R*[*F* ^2^ > 2σ(*F* ^2^)] = 0.033
*wR*(*F* ^2^) = 0.095
*S* = 1.08
1978 reflections
144 parameters
H atoms treated by a mixture of independent and constrained refinement
Δρ_max_ = 0.39 e Å^−3^
Δρ_min_ = −0.22 e Å^−3^

### 

Data collection: *APEX2* (Bruker, 2009 ▶); cell refinement: *SAINT* (Bruker, 2009 ▶); data reduction: *SAINT*; program(s) used to solve structure: *SHELXTL* (Sheldrick, 2008 ▶); program(s) used to refine structure: *SHELXTL*; molecular graphics: *SHELXTL*; software used to prepare material for publication: *SHELXTL* and *PLATON* (Spek, 2009 ▶).

## Supplementary Material

Crystal structure: contains datablocks global, I. DOI: 10.1107/S1600536810039395/is2609sup1.cif

Structure factors: contains datablocks I. DOI: 10.1107/S1600536810039395/is2609Isup2.hkl

Additional supplementary materials:  crystallographic information; 3D view; checkCIF report

## Figures and Tables

**Table 1:** Hydrogen-bond geometry (Å, °)

*D*—H⋯*A*	*D*—H	H⋯*A*	*D*⋯*A*	*D*—H⋯*A*
N1—H1*N*1⋯O4^i^	0.874 (18)	1.944 (18)	2.8112 (12)	171.6 (15)
O1—H1*O*1⋯O4^ii^	0.86 (3)	1.83 (3)	2.6794 (16)	169 (2)
C2—H2*A*⋯O3^iii^	0.93	2.53	3.106 (2)	120
C2—H2*A*⋯O3^i^	0.93	2.31	3.0247 (14)	133
C8—H8*A*⋯O2^ii^	0.93	2.40	3.249 (2)	152

Symmetry codes: (i) 
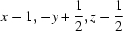
; (ii) 
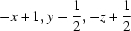
; (iii) 
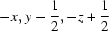
.

**Fig. 1.**
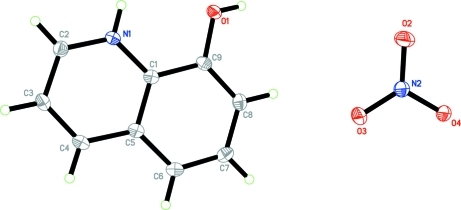
The molecular structure of the title compound showing 50% probability displacement ellipsoids for non-H atoms and the atom-numbering scheme.

**Fig. 2.**
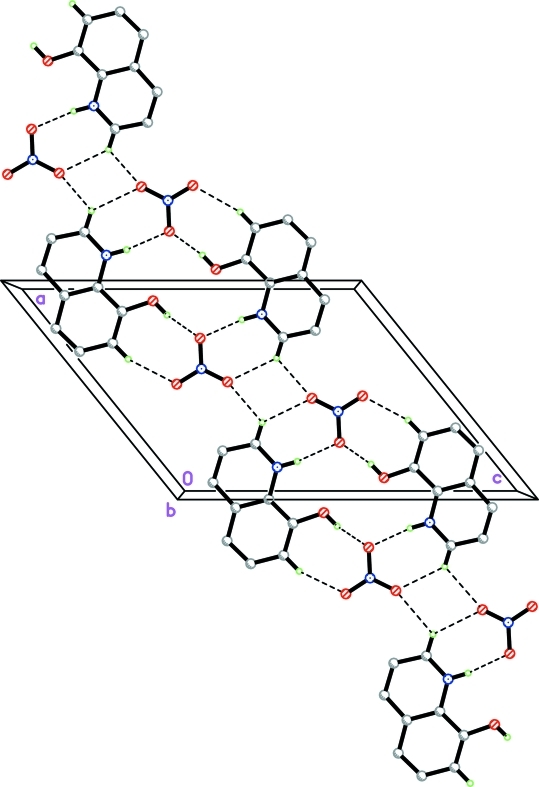
The crystal structure of the title compound, viewed along the b axis.

## supplementary crystallographic information

### Comment

Recently, hydrogen-bonding patterns involving quinoline and its derivatives with organic acid have been investigated (Loh *et al.*, 2010*a*,*b*,*c*,*d*). Syntheses of the quinoline derivatives were discussed earlier (Sasaki *et al.*, 1998; Reux *et al.*, 2009). Quinolines and their derivatives are very important compounds because of their wide occurrence in natural products (Morimoto *et al.*, 1991; Michael, 1997) and biologically active compounds (Markees *et al.*, 1970; Campbell *et al.*, 1988). Herein we report the synthesis of 8-hydroxyquinolin-1-ium nitrate.

The asymmetric unit of the title compound (Fig. 1) consists of one 8-hydroxyquinolin-1-ium cation (C1–C10/N1/N2) and one nitrate anion (O2–O4/N2). One proton is transferred from the hydroxyl group of nitric acid to the atom N1 of 8-hydroxyquinoline during the crystallization, resulting in the formation of salt. The quinoline ring system (C1–C9/N1) is approximately planar with a maximum deviation of 0.043 (1) Å at atom C4. Bond lengths (Allen *et al.*, 1987) and angles are within the normal ranges and are comparable to the related structures (Loh *et al.*, 2010**a*,b,*c*,d*).

In the crystal packing (Fig. 2), *R*_2_^2^(7) ring motifs are formed by intermolecular N1—H1N1···O4 and C2—H2A···O3 hydrogen bonds (Table 1). In addition, pairs of intermolecular O1—H1O1···O4 and C8—H8A···O2 hydrogen bonds (Table 1) link the cations and anions together to generate another set of *R*_2_^2^(8) ring motifs. These sets of ring motifs are further linked into ribbons along the *a* axis *via* intermolecular C2—H2A···O3 hydrogen bonds (Table 1).

### Experimental

A few drops of nitric acid were added to a hot methanol solution (20 ml) of 8-hydroxyquinoline (29 mg, Merck) which had been warmed over a magnetic stirrer hotplate for a few minutes. The resulting solution was allowed to cool slowly to room temperature. Crystals of the title compound appeared after a few days.

### Refinement

Atoms H1N1 and H1O1 were located from the difference Fourier map and were refined freely [N—H = 0.874 (18) Å and O—H = 0.86 (2) Å]. The remaining H atoms were positioned geometrically with the bond length of C—H being 0.93 Å and were refined using a riding model, with *U*_iso_(H) = 1.2*U*_eq_(C).

### Crystal data

**Table:**

C_9_H_8_NO^+^·NO_3_^−^	*F*(000) = 432
*M**_r_* = 208.17	*D*_x_ = 1.602 Mg m^−^^3^
Monoclinic, *P*2_1_/*c*	Mo *K*α radiation, λ = 0.71073 Å
Hall symbol: -P 2ybc	Cell parameters from 5491 reflections
*a* = 11.3186 (2) Å	θ = 3.5–37.4°
*b* = 6.7568 (1) Å	µ = 0.13 mm^−^^1^
*c* = 14.5006 (2) Å	*T* = 100 K
β = 128.882 (1)°	Block, colourless
*V* = 863.27 (2) Å^3^	0.33 × 0.21 × 0.15 mm
*Z* = 4	

### Data collection

**Table:**

Bruker SMART APEXII CCD area-detector diffractometer	1978 independent reflections
Radiation source: fine-focus sealed tube	1754 reflections with *I* > 2σ(*I*)
graphite	*R*_int_ = 0.023
φ and ω scans	θ_max_ = 27.5°, θ_min_ = 3.5°
Absorption correction: multi-scan (*SADABS*; Bruker, 2009)	*h* = −14→14
*T*_min_ = 0.959, *T*_max_ = 0.981	*k* = −8→8
9590 measured reflections	*l* = −18→18

### Refinement

**Table:**

Refinement on *F*^2^	Primary atom site location: structure-invariant direct methods
Least-squares matrix: full	Secondary atom site location: difference Fourier map
*R*[*F*^2^ > 2σ(*F*^2^)] = 0.033	Hydrogen site location: inferred from neighbouring sites
*wR*(*F*^2^) = 0.095	H atoms treated by a mixture of independent and constrained refinement
*S* = 1.08	*w* = 1/[σ^2^(*F*_o_^2^) + (0.0472*P*)^2^ + 0.3584*P*] where *P* = (*F*_o_^2^ + 2*F*_c_^2^)/3
1978 reflections	(Δ/σ)_max_ < 0.001
144 parameters	Δρ_max_ = 0.39 e Å^−^^3^
0 restraints	Δρ_min_ = −0.22 e Å^−^^3^

### Special details

**Table:**

Experimental. The crystal was placed in the cold stream of an Oxford Cryosystems Cobra open-flow nitrogen cryostat (Cosier & Glazer, 1986) operating at 100.0 (1) K.
Geometry. All e.s.d.'s (except the e.s.d. in the dihedral angle between two l.s. planes) are estimated using the full covariance matrix. The cell e.s.d.'s are taken into account individually in the estimation of e.s.d.'s in distances, angles and torsion angles; correlations between e.s.d.'s in cell parameters are only used when they are defined by crystal symmetry. An approximate (isotropic) treatment of cell e.s.d.'s is used for estimating e.s.d.'s involving l.s. planes.
Refinement. Refinement of *F*^2^ against ALL reflections. The weighted *R*-factor *wR* and goodness of fit *S* are based on *F*^2^, conventional *R*-factors *R* are based on *F*, with *F* set to zero for negative *F*^2^. The threshold expression of *F*^2^ > σ(*F*^2^) is used only for calculating *R*-factors(gt) *etc*. and is not relevant to the choice of reflections for refinement. *R*-factors based on *F*^2^ are statistically about twice as large as those based on *F*, and *R*- factors based on ALL data will be even larger.

### Fractional atomic coordinates and isotropic or equivalent isotropic displacement parameters (Å^2^)

**Table:**

	*x*	*y*	*z*	*U*_iso_*/*U*_eq_	
O1	0.07908 (9)	0.09986 (13)	0.12784 (7)	0.0167 (2)	
N1	−0.13600 (11)	0.10475 (14)	0.15595 (8)	0.0139 (2)	
C1	0.01506 (13)	0.11333 (16)	0.25398 (10)	0.0138 (2)	
C2	−0.24612 (13)	0.10646 (17)	0.16438 (10)	0.0163 (2)	
H2A	−0.3465	0.0953	0.0961	0.020*	
C3	−0.21344 (13)	0.12476 (17)	0.27446 (10)	0.0171 (2)	
H3A	−0.2913	0.1279	0.2795	0.021*	
C4	−0.06498 (13)	0.13802 (17)	0.37464 (10)	0.0158 (2)	
H4A	−0.0424	0.1546	0.4479	0.019*	
C5	0.05450 (13)	0.12672 (16)	0.36803 (10)	0.0144 (2)	
C6	0.21000 (13)	0.12812 (17)	0.46913 (10)	0.0171 (2)	
H6A	0.2383	0.1339	0.5447	0.020*	
C7	0.31841 (13)	0.12080 (18)	0.45453 (10)	0.0186 (3)	
H7A	0.4204	0.1209	0.5210	0.022*	
C8	0.27878 (13)	0.11312 (18)	0.34078 (11)	0.0177 (2)	
H8A	0.3547	0.1114	0.3334	0.021*	
C9	0.12859 (13)	0.10813 (16)	0.24054 (10)	0.0148 (2)	
O2	0.52824 (10)	0.47945 (18)	0.24518 (8)	0.0321 (3)	
O3	0.53907 (10)	0.42125 (15)	0.39694 (8)	0.0239 (2)	
O4	0.74765 (9)	0.44023 (13)	0.41965 (7)	0.0179 (2)	
N2	0.60151 (11)	0.44742 (16)	0.35208 (9)	0.0177 (2)	
H1N1	−0.1627 (19)	0.091 (2)	0.0852 (16)	0.028 (4)*	
H1O1	0.145 (2)	0.056 (3)	0.1211 (17)	0.042 (5)*	

### Atomic displacement parameters (Å^2^)

**Table:**

	*U*^11^	*U*^22^	*U*^33^	*U*^12^	*U*^13^	*U*^23^
O1	0.0159 (4)	0.0230 (4)	0.0140 (4)	0.0024 (3)	0.0108 (3)	0.0012 (3)
N1	0.0159 (5)	0.0145 (5)	0.0124 (4)	0.0001 (4)	0.0095 (4)	−0.0002 (3)
C1	0.0161 (5)	0.0114 (5)	0.0137 (5)	0.0003 (4)	0.0094 (4)	0.0003 (4)
C2	0.0153 (5)	0.0169 (5)	0.0167 (5)	−0.0007 (4)	0.0100 (4)	−0.0008 (4)
C3	0.0189 (5)	0.0183 (6)	0.0200 (5)	−0.0007 (4)	0.0150 (5)	−0.0012 (4)
C4	0.0216 (6)	0.0138 (5)	0.0150 (5)	0.0000 (4)	0.0130 (5)	−0.0003 (4)
C5	0.0183 (5)	0.0114 (5)	0.0146 (5)	−0.0001 (4)	0.0108 (5)	0.0000 (4)
C6	0.0198 (5)	0.0163 (5)	0.0133 (5)	0.0006 (4)	0.0095 (5)	−0.0004 (4)
C7	0.0152 (5)	0.0191 (6)	0.0160 (5)	0.0010 (4)	0.0071 (4)	−0.0004 (4)
C8	0.0163 (5)	0.0186 (6)	0.0203 (6)	0.0008 (4)	0.0126 (5)	0.0003 (4)
C9	0.0184 (5)	0.0132 (5)	0.0147 (5)	0.0010 (4)	0.0113 (5)	0.0010 (4)
O2	0.0186 (4)	0.0607 (7)	0.0157 (4)	0.0030 (4)	0.0101 (4)	0.0078 (4)
O3	0.0169 (4)	0.0395 (5)	0.0197 (4)	−0.0012 (4)	0.0137 (4)	0.0007 (4)
O4	0.0121 (4)	0.0260 (5)	0.0155 (4)	0.0000 (3)	0.0087 (3)	−0.0005 (3)
N2	0.0141 (4)	0.0238 (5)	0.0159 (5)	0.0002 (4)	0.0098 (4)	−0.0006 (4)

### Geometric parameters (Å, °)

**Table:**

O1—C9	1.3558 (13)	C4—H4A	0.9300
O1—H1O1	0.86 (2)	C5—C6	1.4170 (15)
N1—C2	1.3266 (15)	C6—C7	1.3707 (16)
N1—C1	1.3770 (14)	C6—H6A	0.9300
N1—H1N1	0.874 (18)	C7—C8	1.4126 (16)
C1—C9	1.4160 (16)	C7—H7A	0.9300
C1—C5	1.4196 (15)	C8—C9	1.3790 (16)
C2—C3	1.3995 (16)	C8—H8A	0.9300
C2—H2A	0.9300	O2—N2	1.2343 (13)
C3—C4	1.3701 (16)	O3—N2	1.2372 (13)
C3—H3A	0.9300	O4—N2	1.2899 (12)
C4—C5	1.4166 (16)		
			
C9—O1—H1O1	114.7 (13)	C4—C5—C1	117.82 (10)
C2—N1—C1	122.28 (10)	C6—C5—C1	118.93 (10)
C2—N1—H1N1	117.2 (11)	C7—C6—C5	119.40 (10)
C1—N1—H1N1	120.5 (11)	C7—C6—H6A	120.3
N1—C1—C9	120.19 (10)	C5—C6—H6A	120.3
N1—C1—C5	118.95 (10)	C6—C7—C8	121.52 (11)
C9—C1—C5	120.86 (10)	C6—C7—H7A	119.2
N1—C2—C3	120.99 (10)	C8—C7—H7A	119.2
N1—C2—H2A	119.5	C9—C8—C7	120.63 (11)
C3—C2—H2A	119.5	C9—C8—H8A	119.7
C4—C3—C2	119.03 (10)	C7—C8—H8A	119.7
C4—C3—H3A	120.5	O1—C9—C8	125.09 (10)
C2—C3—H3A	120.5	O1—C9—C1	116.28 (10)
C3—C4—C5	120.81 (10)	C8—C9—C1	118.62 (10)
C3—C4—H4A	119.6	O2—N2—O3	122.01 (10)
C5—C4—H4A	119.6	O2—N2—O4	119.39 (9)
C4—C5—C6	123.24 (10)	O3—N2—O4	118.60 (9)
			
C2—N1—C1—C9	179.06 (10)	C4—C5—C6—C7	−178.64 (11)
C2—N1—C1—C5	−0.72 (16)	C1—C5—C6—C7	1.38 (16)
C1—N1—C2—C3	2.44 (17)	C5—C6—C7—C8	0.40 (18)
N1—C2—C3—C4	−0.98 (17)	C6—C7—C8—C9	−1.52 (18)
C2—C3—C4—C5	−2.14 (17)	C7—C8—C9—O1	−179.71 (11)
C3—C4—C5—C6	−176.26 (11)	C7—C8—C9—C1	0.77 (17)
C3—C4—C5—C1	3.72 (16)	N1—C1—C9—O1	1.69 (15)
N1—C1—C5—C4	−2.31 (15)	C5—C1—C9—O1	−178.53 (10)
C9—C1—C5—C4	177.91 (10)	N1—C1—C9—C8	−178.75 (10)
N1—C1—C5—C6	177.67 (10)	C5—C1—C9—C8	1.03 (16)
C9—C1—C5—C6	−2.11 (16)		

### Hydrogen-bond geometry (Å, °)

**Table:**

*D*—H···*A*	*D*—H	H···*A*	*D*···*A*	*D*—H···*A*
N1—H1N1···O4^i^	0.874 (18)	1.944 (18)	2.8112 (12)	171.6 (15)
O1—H1O1···O4^ii^	0.86 (3)	1.83 (3)	2.6794 (16)	169 (2)
C2—H2A···O3^iii^	0.93	2.53	3.106 (2)	120
C2—H2A···O3^i^	0.93	2.31	3.0247 (14)	133
C8—H8A···O2^ii^	0.93	2.40	3.249 (2)	152

Symmetry codes: (i) *x*−1, −*y*+1/2, *z*−1/2; (ii) −*x*+1, *y*−1/2, −*z*+1/2; (iii) −*x*, *y*−1/2, −*z*+1/2.
